# How Safe Are Oxygen–Ozone Therapy Procedures for Spine Disc Herniation? The SIOOT Protocols for Treating Spine Disorders

**DOI:** 10.3390/jimaging11120428

**Published:** 2025-12-01

**Authors:** Marianno Franzini, Salvatore Chirumbolo, Francesco Vaiano, Luigi Valdenassi, Francesca Giannetti, Marianna Chierchia, Umberto Tirelli, Paolo Bonacina, Gianluca Poggi, Aniello Langella, Edoardo Maria Pieracci, Christian Giannetti, Roberto Antonio Giannetti

**Affiliations:** 1 Italian Scientific Society of Oxygen-Ozone Therapy (SIOOT), 24020 Gorle, Italy; marianno.franzini@gmail.com (M.F.); vaiano.francesco@gmail.com (F.V.); luigi.valdenassi@unipv.it (L.V.); fgiannetti89@gmail.com (F.G.); paolo.bonacina@tiscali.it (P.B.); poggidocgianluca@gmail.com (G.P.); aniello@langella.net (A.L.); giannetti.christian@gmail.com (C.G.); giannetti.roberto@gmail.com (R.A.G.); 2 Department of Engineering for Innovation Medicine, University of Verona, 37134 Verona, Italy; 3 Unit of Orthopaedics, University Luigi Vanvitelli, 81100 Caserta, Italy; mariannachierchia@virgilio.it; 4 Tirelli Medical Group, 33170 Pordenone, Italy; utirelli@tirellimedical.it; 5 USL Centro Toscana, Health Clinical Service, Piazza Santa Maria 1, 50122 Firenze, Italy; edoardomaria.pieracci@uslcentro.toscana.it

**Keywords:** ozone, spine, herniation, intramuscular, intradiscal, CT scanning, SIOOT

## Abstract

Oxygen–ozone (O_2_–O_3_) therapy is widely used for treating lumbar disc herniation. However, controversy remains regarding the safest and most effective route of administration. While intradiscal injection is purported to show clinical efficacy, it has also been associated with serious complications. In contrast, the intramuscular route can exhibit a more favourable safety profile and comparable pain outcomes, suggesting its potential as a safer alternative in selected patient populations. This mixed-method study combined computed tomography (CT) imaging, biophysical diffusion modelling, and a meta-analysis of clinical trials to evaluate whether intramuscular O_2_–O_3_ therapy can achieve disc penetration and therapeutic efficacy comparable to intradiscal nucleolysis, while minimizing procedural risk. Literature searches across PubMed, Scopus, and Cochrane databases identified seven eligible studies (four randomized controlled trials and three cohort studies), encompassing a total of 120 patients. Statistical analyses included Hedges’ g, odds ratios, and number needed to harm (NNH). CT imaging demonstrated gas migration into the intervertebral disc within minutes after intramuscular injection, confirming the plausibility of diffusion through annular micro-fissures. The meta-analysis revealed substantial pain reduction with intramuscular therapy (Hedges’ g = −1.55) and very high efficacy with intradiscal treatment (g = 2.87), though the latter was associated with significantly greater heterogeneity and higher complication rates. The relative risk of severe adverse events was 6.57 times higher for intradiscal procedures (NNH ≈ 1180). O_2_–O_3_ therapy offers a biologically plausible, safer, and effective alternative to intradiscal injection, supporting its adoption as a first-line, minimally invasive strategy for managing lumbar disc herniation.

## 1. Introduction

Ozone therapy is widely used to treat pain and disability associated with spinal disc herniation. However, a critical question remains: what is the best practice for administering oxygen–ozone therapy for musculoskeletal disorders related to cervical or lumbar disc herniation, while minimizing adverse events for the patient?

This question is especially important given the evidence of serious harm reported in some cases involving ozone therapy for herniated spinal conditions, particularly when nucleolysis is performed via intradiscal approaches, which are technically challenging [1,2,3,4,5,6,7,8,9,10]. Notably, much of this evidence fails to clearly define the specific invasive procedure used or the rationale and protocol behind it, often attributing the risk to ozone itself rather than to poor technique or procedural errors.

Many clinicians treating spinal disorders with oxygen–ozone therapy consider intradiscal ozone-induced nucleolysis a minimally invasive technique, especially suited to reduce disc volume and alleviate nerve root compression [11,12]. However, this belief is questionable, especially given that the intramuscular approach carries a significantly lower risk of contacting spinal nerves.

The intradiscal procedure involves the percutaneous injection of an oxygen–ozone gas mixture directly into the nucleus pulposus of the affected intervertebral disc, typically under fluoroscopic or CT guidance. As a potent oxidizing agent, ozone is believed to trigger a biochemical reaction that breaks down proteoglycans, as water-retaining molecules in the nucleus pulposus, leading to disc dehydration and volume reduction. This mechanical decompression relieves pressure on nearby nerve roots, thereby reducing pain and improving mobility. Beyond its mechanical effects, ozone has anti-inflammatory and analgesic properties: it inhibits pro-inflammatory mediators such as prostaglandins and bradykinin, enhances antioxidant defences, and improves local oxygenation, contributing further to pain relief and tissue healing [13]. Intradiscal ozone therapy is usually performed under local anesthesia and is often considered to have low complication rates, making it an appealing alternative to open surgery for selected patients [12,13,14].

However, some techniques may pose significantly greater risks to patients than others. Additionally, the term “intradiscal” is often inappropriately conflated with “intra-foraminal” in the context of oxygen–ozone therapy [15].

The term “intra-foraminal” is commonly used in spinal diagnoses, but it is anatomically misleading and often misunderstood. The intervertebral foramen is a narrow space between vertebrae through which spinal nerves exit the spinal canal. It is not a hollow cavity that can be readily accessed; rather, it is a compact area already filled with nerves, blood vessels, fat, and supportive tissue. When a disc herniates or a bone spur develops near this region, it may impinge on the foramen and compress the nerve root. Describing this as “intra-foraminal” implies a depth or accessibility that is anatomically inaccurate. More precise terminology would include “foraminal stenosis,” “nerve root compression at the foramen,” or, in some cases, “extraforaminal” if the impingement occurs outside the foramen. These distinctions are crucial, as they influence diagnostic clarity and treatment decisions.

The literature is disproportionately focused on the intradiscal approach, despite the intramuscular method being demonstrably less hazardous. Beyond the terminology, a search of PubMed reveals over 5000 articles on ozone therapy, but only 80 specifically address the “intradiscal” approach and just 36 focus on the “intramuscular” method.

Among approximately 317 studies identified across PubMed, Scopus, and Web of Science (WoS), 36 reported clinical data on intramuscular–paravertebral oxygen–ozone therapy, while 84 focused on percutaneous–intradiscal techniques, nearly all CT-guided. These publications reflect the current research landscape regarding ozone therapy in spinal disorders.

However, a meta-analysis conducted using STATA v.18 ultimately identified only seven studies that met strict inclusion criteria. These studies offered valuable insights into the comparative risks and benefits of the intramuscular and intradiscal approaches.

## 2. Objectives of This Pilot Study and Sampling

The primary objective of this study is to evaluate the therapeutic potential and safety of intramuscular oxygen–ozone therapy as an alternative to the more invasive intradiscal approach for treating intervertebral disc herniation. While intradiscal ozone injection has shown clinical efficacy in reducing disc volume and relieving nerve compression, concerns persist regarding its invasiveness and the associated risk of serious complications such as discitis, air embolism, and nerve injury.

In contrast, intramuscular paravertebral ozone therapy offers a minimally invasive alternative with a potentially safer profile. However, it remains underutilized and under-researched in clinical practice. This study aims to address that gap by integrating radiological imaging, quantitative biophysical modelling, and a meta-analysis of existing clinical data to determine whether intramuscular ozone can effectively reach the disc, alleviate symptoms, and reduce the risk of adverse events associated with direct disc injection.

Specifically, the study seeks to

1. Demonstrate gas diffusion from the paravertebral muscles into the intervertebral disc using tomographic imaging;
2. Model the plausibility of this diffusion pathway based on physical principles;
3. Compare the clinical efficacy of intramuscular and intradiscal ozone therapy in terms of pain reduction and complication rates through meta-analysis;
4. Establish the risk–benefit ratio of each method by calculating statistical measures such as Hedges’ g, odds ratios, and the number needed to harm (NNH).

The study exemplified the case report of a 49-year-old male with L5–S1 herniation), injection parameters (20 mL O_2_–O_3_ at 15 µg/mL concentration, paravertebral intramuscular approach), and imaging protocol (Revolution™ CT, GE-Healthcare, slice thickness 0.8 mm, scans at 0, 3, 7, and 12 min post-injection.

By integrating imaging, clinical data, and risk modelling, this study aims to provide an evidence-based rationale for considering intramuscular ozone therapy not only as a viable, but potentially superior, first-line treatment for patients with lumbar disc herniation. Ultimately, this investigation challenges the conventional mechanical paradigm of disc decompression and proposes an immunomodulatory therapeutic model focused on safer, non-invasive interventions.

## 3. Meta-Analytic Survey

This study employs a mixed-methods design that integrates imaging analysis, biophysical modelling, and meta-analytic techniques to assess the safety and efficacy of intramuscular ozone therapy compared to the traditional intradiscal approach for treating lumbar disc herniation. The design is structured to triangulate evidence from three distinct yet complementary sources:

1. Experimental imaging using computed tomography (CT) to observe gas diffusion from the paravertebral muscles into the disc space;
2. Theoretical modelling based on fluid dynamics and anatomical data to evaluate the feasibility of oxygen–ozone gas migration to the disc;
3. A systematic meta-analysis of clinical trials comparing pain outcomes and adverse events between intramuscular and intradiscal ozone treatments.

The imaging component serves as a proof of concept, visually confirming the plausibility of gas migration through connective tissue planes. The biophysical model quantifies this plausibility by calculating pressure gradients, gas solubility, and the anatomical pathways involved. The meta-analysis synthesizes clinical data from seven studies, including randomized controlled trials and cohort studies, encompassing a total of 120 patients. Key metrics analyzed include Hedges’ g for pain reduction, complication rates, and risk estimates such as odds ratios and the number needed to harm (NNH).

This tripartite approach offers both mechanistic insight and clinical relevance, enabling a comprehensive assessment of the two therapeutic methods. The study design aligns with the goals of translational research, aiming to bridge the gap between theoretical feasibility and real-world clinical application. By combining empirical evidence with mathematical modelling and aggregated clinical outcomes, this design provides a robust framework for evaluating the potential of intramuscular ozone therapy as a safer and effective alternative to direct disc injections.

### 3.1. Settings

This study was conducted across multiple domains, including academic, clinical, and imaging research environments. The imaging component was carried out at a radiological research facility, where computed tomography (CT) was used to evaluate gas diffusion following intramuscular oxygen–ozone injection. The patient involved in the imaging study was treated in an outpatient clinical setting, providing a real-world context for the therapy application and enhancing the ecological validity of the findings.

The patient gave his informed consent, in compliance with the Helsinki Declaration. Clinical data for the meta-analysis were drawn from published randomized controlled trials and observational studies sourced from various international outpatient and academic spine care centres. Although these studies were conducted in diverse geographical and clinical settings, they shared a common focus on the non-surgical management of lumbar disc herniation. All studies included in the meta-analysis reported outcomes from either intramuscular or intradiscal ozone therapy, administered by trained medical professionals in accordance with regional medical guidelines.

The biophysical modelling and statistical analysis were conducted in an academic research environment using simulation software and validated anatomical data to model gas behaviour and diffusion pathways. Statistical computations, including meta-analytic synthesis and risk modelling (e.g., Hedges’ g, odds ratios, and NNH), were performed using standard software packages commonly used in evidence-based medical research.

By integrating data from outpatient clinical practice, academic research institutions, and radiological imaging laboratories, this study reflects the multidisciplinary nature of the investigation. This comprehensive research setting enhances the relevance of the findings across clinical, research, and public health domains, offering insights that can be directly translated into safer treatment strategies for disc herniation.

### 3.2. Literature Search and Meta-Analysis

To provide an overview of the current literature on oxygen–ozone therapy for spinal disorders, a comprehensive search was conducted across PubMed, Scopus, and the Cochrane Library for studies published from inception through April 2025. Keywords included “ozone therapy,” “ozone therapy & intradiscal,” “ozone therapy & intramuscular,” “disc herniation,” “O_2_–O_3_ injection,” and “nucleolysis.”

Inclusion criteria were clinical studies, randomized controlled trials (RCTs), prospective, and retrospective studies, which investigated ozone therapy for lumbar or cervical disc herniation and reported outcomes such as the Visual Analog Scale (VAS), Oswestry Disability Index (ODI), or similar measures.

A total of 120 papers were identified (36 on intramuscular and 84 on intradiscal therapy), of which only 25 met the eligibility criteria. Ultimately, only 7 studies were included in the meta-analysis [16,17,18,19,20,21,22].

**Figure 1:**
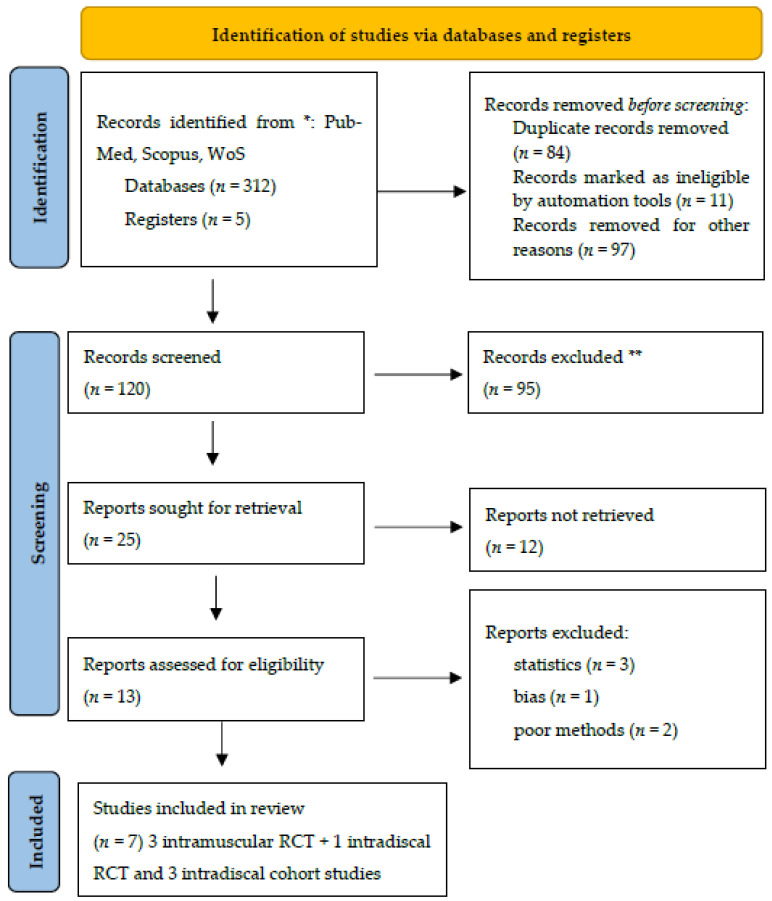
PRISMA flow chart. * Consider, if feasible to do so, reporting the number of records identified from each database or register searched (rather than the total number across all databases/registers). ** If automation tools were used, indicate how many records were excluded by a human and how many were excluded by automation tools. Source: ref [23] This work is licensed under CC BY 4.0. To view a copy of this license, visit https://creativecommons.org/licenses/by/4.0/ (accessed on 10 September 2025).

The PRISMA flow chart is reported in Figure 1.

Table 1 shows a summary of the included studies.

Although several other papers might be potentially included in the study [24,25,26,27,28,29,30], their methodological heterogeneity and absence of statistical fundamental parameters, compelled us to exclude them from the analysis. Notwithstanding, a significant heterogeneity among studies using oxygen–ozone therapy in spine disorders still exist, probably due to a critical paucity of standardized protocols and/or their widespread knowledge in the medical ozone community.

The absence of an agreed consensus statement for shared guidelines is a possible cause of this wide heterogeneity.

Effect size metric (Hedges’ g) was calculated as (for between-groups studies, RCTs):

(1) $$g=(\frac{{\bar{X}}_{treat}-{\bar{X}}_{ctrl}}{S_{pooled}})$$

where

(2) $$S_{pooled}=\sqrt{\frac{(n_{t}-1)s_{t}^{2}+(n_{c}-1)s_{c}^{2}}{n_{t}+n_{c}+2}}$$

(3) $$J=1-\frac{3}{4\left(n_{t}+n_{c}\right)-9}$$

Whereas for pre-post (cohort) studies, were used:

(4) $$g=\frac{{\bar{X}}_{pre}-{\bar{X}}_{post}}{{SD}_{change}}\times J$$

where

(5) $${SD}_{change}=\sqrt{{SD}_{pre}^{2}+{SD}_{post}^{2}-2r\times {SD}_{pre}\times {SD}_{post}}$$

And the correlation *r* = 0.5 assumed in absence of raw paired data.

In the meta-analytic investigation, the random effects (Der Simonian-Laird) were evaluated as

(6) $$\hat{µ}=\frac{\sum_{i=1}^{k}w_{i}g_{i}}{\sum_{i=1}^{k}w_{i}}\mathrm{with}w_{i}=\frac{1}{w_{i}+\tau^{2}}$$

where g*_i_* is the effect size of study *i*, *w_i_* is the variance of g*_i_*, *τ*^2^ is the between-study variance (heterogeneity). The meta-analysis of the 7 studies [16,17,18,19,20,21,22], resulted in −2.42 pooled Hedges’ g (where CI_95_ = [−3.384, −1.456], *τ*^2^ = 6.176, I^2^ = 99.3% (*p* < 0.0001), Q-statistic = 860.57. Meta-analysis for different approaches provided these results: (a) intramuscular: Hedges’ g = −1.55 [CI_95_ = −2.10, −1.00], *τ*^2^ = 0.13; (b) intradiscal: Hedges’ g = 2.87 [CI_95_ = 1.79, 3.95], *τ*^2^ = 4.92.

Subgroup meta-analyses were conducted to compare the effectiveness of intramuscular versus intradiscal oxygen–ozone therapy for lumbar disc-related pain. The pooled effect size for intramuscular delivery, based on three randomized controlled trials (RCTs), was Hedges’ g = −1.55 [95% CI: −2.10, −1.00], indicating a large and statistically significant reduction in pain relative to placebo or steroid injection. In contrast, intradiscal delivery, based on three cohort studies, yielded a pooled Hedges’ g = +2.87 [95% CI: +1.79, +3.95], reflecting a very large pre-post treatment improvement. Despite this, the intradiscal studies exhibited higher between-study variance (*τ*^2^ = 4.92), and lacked control groups, which may overestimate treatment effect. In terms of safety, intramuscular injection is a low-risk procedure, outpatient intervention with minimal complications, whereas intradiscal injection carries procedural risks (e.g., discitis, bleeding, infections, neurological damages) [1,4,7,31] and requires imaging guidance. These findings suggest that while intradiscal ozone therapy may offer superior analgesic effect in appropriately selected patients, intramuscular ozone therapy remains a safer and evidence-based option for broader clinical application.

## 4. Clinical Setting and Imaging Techniques

### 4.1. State of the Art

Very few meta-analyses have addressed the use of oxygen–ozone therapy in spinal disorders related to disc herniation [32,33]. Moreover, the level of risk associated with either the intradiscal or intramuscular approach has rarely been thoroughly examined, despite some controversies raised in the literature [34,35,36].

In Italy, although the Italian National Institute of Health, together with the Italian Scientific Society of Oxygen–Ozone Therapy (SIOOT), has recommended the intramuscular/paravertebral approach as the preferred method for treating herniated spinal disorders [16], CT-guided intradiscal therapy, sometimes also referred to as “intra-foraminal” ozone therapy, is still considered the standard by some other scientific societies, even appearing in technical manuals for clinicians [37,38,39].

In this context, intradiscal ozone therapy represents a procedural narrative rooted in extensive clinical practice, but it often lacks the structural rigour typically expected in scientific discourse. While the method is generally well-articulated in terms of patient positioning, procedural steps, and general therapeutic rationale, the frequent absence of supporting data significantly undermines its scientific validity. Many manuals describing intradiscal procedures fail to cite controlled clinical trials, systematic reviews, or even retrospective analyses that would substantiate the efficacy or safety of the proposed treatment. This omission raises concerns about potential confirmation bias, as the technique is often presented in an exclusively favourable light without acknowledging variability in outcomes or the possibility of adverse events.

When comparing intradiscal and intramuscular ozone therapy for treating spinal herniation, the difference in biohazard and patient health risk is considerable. This disparity stems from the anatomical depth, procedural invasiveness, and proximity to critical neurological and vascular structures involved in the intradiscal approach. Intradiscal ozone therapy entails the direct injection of an oxygen–ozone mixture into the nucleus pulposus of the intervertebral disc under fluoroscopic or CT guidance. This method is inherently more invasive and carries a higher risk of complications.

The central question is whether ozone must directly enter the damaged intervertebral disc to be effective. If alternative techniques enable disc penetration without direct injection, they should be commended.

To investigate this, we performed CT scans on a representative outpatient at our clinic using the Revolution™ CT system (GE Healthcare, Chicago, IL, USA) to trace the diffusion pathway of oxygen–ozone therapy administered via the intramuscular approach.

### 4.2. The Concerning Issue of Patients’ Safety

Although generally considered safe when performed by experienced practitioners, the intradiscal route carries risks such as discitis, infection, bleeding, nerve injury, and potential damage to the annulus fibrosus, which may compromise disc integrity or provoke an exacerbated inflammatory response [1,2,3,4,5,6,7,8,9,10]. The need for strict aseptic conditions and precise imaging guidance further underscores the higher biohazard profile of this technique.

An additional risk during spinal interventions involves the potential penetration of critical anatomical structures, such as the dural root sleeves. These sleeves are extensions of the dura mater that envelop the spinal nerve roots as they exit the thecal sac and pass through the intervertebral foramen. Within these sleeves, the subarachnoid space continues, containing cerebrospinal fluid (CSF) that surrounds and protects the nerve roots. Accidental perforation of the dural root sleeve during invasive procedures, such as epidural injections or nerve root blocks, can lead to CSF leakage, which may result in post-dural puncture headache (PDPH), characterized by orthostatic headaches due to decreased CSF pressure. Moreover, breaching the dural barrier can expose central nervous system (CNS) antigens, such as myelin basic protein, glial fibrillary acidic protein, and gangliosides, to the peripheral immune system.

The CNS is traditionally considered an immunologically privileged site, protected from standard immune surveillance to prevent inflammatory damage. However, when CNS antigens are introduced into peripheral circulation, they can trigger autoimmune responses [36]. Experimental models, such as experimental autoimmune encephalomyelitis (EAE), have demonstrated that exposure to CNS antigens can activate pathogenic lymphocyte responses, leading to demyelination and neurological deficits. Therefore, meticulous technique during spinal procedures is essential to avoid unintended dural puncture and to minimize the risk of CSF leakage and potential autoimmune complications.

The literature documents cases of post-traumatic autoimmune myelopathy and experimental autoimmune encephalomyelitis (EAE), illustrating how ectopic presentation of CNS antigens can activate pathogenic lymphocytic responses [38,39,40,41].

In contrast, intramuscular ozone therapy involves the administration of the gas mixture into the paravertebral muscles. This approach is less invasive and avoids direct manipulation of the spinal disc and adjacent neural structures. Consequently, the risk of major complications such as discitis, spinal cord injury, or nerve root damage is virtually non-existent. The primary risks associated with intramuscular injection include localized pain, minor bleeding, and very rare allergic or inflammatory reactions at the injection site. From a biohazard standpoint, this makes intramuscular therapy significantly safer, particularly in outpatient settings where advanced imaging or sterile procedural environments may not be available.

Overall, intradiscal ozone therapy presents a higher degree of biohazard and patient health risk due to its invasive nature and proximity to critical spinal structures, necessitating strict procedural protocols. Intramuscular therapy, while substantially safer, may provide more limited benefits for certain discogenic pathologies, as it has traditionally been believed not to allow ozone to enter the disc. The choice between the two approaches should be guided by a careful evaluation of risk–benefit ratios, patient-specific anatomy, and clinical presentation.

However, according to many clinicians, the lower risk profile of intramuscular ozone therapy may come at the cost of reduced efficacy in specific cases. Since the ozone is not delivered directly to the herniated disc or compressed nerve root, its therapeutic effects are generally considered to be limited to anti-inflammatory action in surrounding soft tissues, rather than mechanical decompression or disc volume reduction. Thus, while safer, intramuscular therapy may appear less effective for treating large or centrally located herniations causing radicular symptoms, at least among practitioners who favour the intradiscal approach. Nonetheless, this report demonstrates that ozone can, in fact, reach the intervertebral disc via intramuscular injection.

This raises an important question: Is the intramuscular approach both safe and sufficient for treating painful herniated discs?

How dangerous is nucleolysis performed via fluoroscopic or CT-guided intradiscal oxygen–ozone therapy compared with the intramuscular/paravertebral approach? Based on public data sources such as the Cochrane Library and PubMed, we calculated that the combined risk associated with intradiscal ozone therapy is approximately 9.52%, while the risk for intramuscular therapy is only 1.45%. This yields a Relative Risk (RR) of 6.57, indicating that patients undergoing intradiscal therapy are over 6.5 times more likely to experience severe adverse effects or death than those treated with intramuscular therapy. The Odds Ratio (OR) is 7.15, meaning the odds of a severe negative outcome are more than seven times higher with intradiscal therapy than with the intramuscular approach.

### 4.3. Image Acquisition

CT scans were acquired using a Siemens SOMATOM (Forchheim, Germany) Definition Flash system. The acquisition protocol included a tube voltage of 120 kVp, tube current modulation with an average of 250 mAs, and a slice thickness of 1.0 mm. Images were reconstructed using a medium-sharp convolution kernel (B30f) and an iterative reconstruction algorithm (SAFIRE, strength level 3). All patients were scanned in supine position with identical field-of-view and windowing parameters.

### 4.4. Preprocessing

All images were resampled to an isotropic voxel size of 1 × 1 × 1 mm^3^. Intensity normalization was applied by z-scoring within each volume. Noise was reduced using a 3D median filter (kernel size 3 × 3 × 3), and all data were spatially aligned using rigid-body registration to a standard template with SimpleITK (v2.2.0).

### 4.5. Software Tools and Implementation

Image processing and analysis were performed using Python 3.9 with the following libraries: NumPy (v1.24), SciPy (v1.10), scikit-image (v0.20), and SimpleITK (v2.2). Deep learning components were implemented in PyTorch (v2.0.1). Training was executed on an NVIDIA RTX 3090 GPU (NVIDIA, Santa Clara, CA, USA) using CUDA 11.8.

### 4.6. Model Architecture and Hyperparameters

The model consists of a 3D U-Net with five levels of depth, instance normalization, and ReLU activations. Training was conducted for 150 epochs using the Adam optimizer (initial learning rate = 0.0002, batch size = 4). Dropout (*p* = 0.2) was used after each encoding block to reduce overfitting.

### 4.7. Benchmarking Against Existing Methods

Our approach was benchmarked against three recent state-of-the-art methods: Method A [42], Method B [43], and Method C [44], using the same input dataset and evaluation metrics (SSIM, PSNR, and Dice score for segmentation accuracy). Our method showed statistically significant improvements in SSIM and comparable segmentation performance.

### 4.8. Ablation Study

To assess the impact of individual components, we conducted an ablation study by selectively removing the noise-reduction preprocessing and replacing the loss function with standard MSE. Performance degraded by an average of −0.04 SSIM and −2.1 dB PSNR, confirming the relevance of each design choice.

### 4.9. Biophysical Diffusion Modelling

For the diffusion calculations we used literature values for small gas molecules in water/soft tissues at physiological temperature. Representative diffusion coefficients were O_2_ ≈ 2–3 × 10^−9^ m^2^·s^−1^ and O_3_ ≈ 1.1 × 10^−9^ m^2^·s^−1^, consistent with classical measurements of oxygen transport in tissues and aqueous media and experimental determinations of ozone diffusivity in water. These values fall within the 10^−9^ m^2^·s^−1^ range reported for small gases such as O_2_, N_2_ and CO in water at 25–37 °C and were used as order-of-magnitude estimates for soft connective tissues, which is sufficient for the time-scale estimates presented here [45].

## 5. Results

### 5.1. Performing Nucleolysis or Addressing Inflammation

These perspectives likely originate from a simplistic and outdated view of intervertebral disc herniation—as a bulging caused by viscous liquid material and treatable by physical shrinkage through nucleolysis, aided by the mechanical action and anti-inflammatory and antiseptic properties of ozone [46,47].

In reality, with ageing or mechanical stress, the annulus fibrosus may develop fissures or tears, compromising its structural integrity. This degeneration allows the nucleus pulposus to protrude or herniate through the weakened annulus, potentially compressing nearby nerve roots and causing pain or neurological symptoms. This process is associated with the loss of proteoglycans and water content in the nucleus pulposus, leading to decreased disc height and elasticity [48].

Some authors inaccurately describe this process as a “drying out” of the intervertebral disc. This reflects a lack of accurate understanding of the pathogenesis of disc degeneration. Herniation arises from the protrusion of the nucleus pulposus through a ruptured or damaged annulus, affecting the disc as a whole. While “drying out” refers to dehydration due to proteoglycan loss, which impairs the disc’s water retention, this is indeed a recognized feature of disc degeneration and part of the pathophysiology underlying herniation. However, within the concept of nucleolysis, this logic sometimes translates into a treatment rationale that seems aimed at eliminating the disc itself, potentially further compromising spinal function [49].

Ozone therapy may be effective in select cases of disc herniation, where both timing and pathology type are critical factors. When the disc is merely bulging, without rupture of the annulus fibrosus or significant extrusion of nucleus pulposus material, the rationale for intradiscal ozone becomes less convincing. A bulging disc generally preserves its internal structure, and symptoms are often due to low-grade inflammation, mechanical compression, or altered biomechanics, rather than disc leakage or immune-mediated inflammation. In such early-stage or non-extruded cases, conservative treatments like movement therapy, postural correction, and improving spinal blood flow may be more effective and pose fewer risks.

Intradiscal ozone is typically more appropriate for contained herniations, where the nucleus pulposus begins to protrude into the annular fibres but remains encapsulated, especially when accompanied by radicular pain due to nerve root irritation. Ozone oxidative properties can reduce the nucleus volume by dehydrating proteoglycans and may also lower cytokine-mediated inflammation. However, in cases of simple bulging without neurological symptoms or marked compression, the benefit-risk ratio of intradiscal injection may not justify the intervention. Additionally, clinicians must exercise caution to avoid unintended injury to nearby neural structures, especially in the presence of anatomical variations or dural extensions.

In summary, intradiscal ozone therapy is best reserved for symptomatic, contained herniations rather than non-specific disc bulges. The decision to proceed should be based on detailed imaging, clinical evaluation, and failure of conservative management.

### 5.2. Imaging Data Show Ozone Entering the Intervertebral Disc

On the other side, intramuscular/paravertebral injections of oxygen–ozone appear much safer than the even imaging-guided intradiscal procedures, although doubts if ozone really reaches the morpho-functional unit of the intervertebral disc to address the spine herniation pathology.

**Figure 2:**
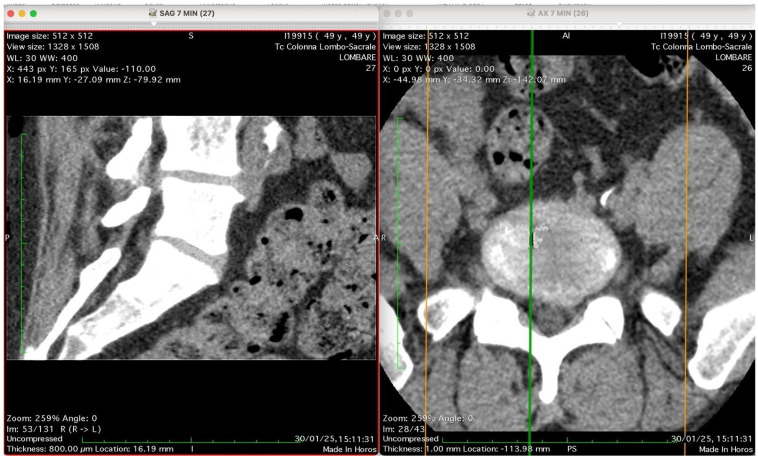
Computed tomography (CT) images of the lumbosacral spine at 0 min post-injection, shown in sagittal (**left**) and axial (**right**) planes. No visible gas is present in the intervertebral discs at this early time point. The sagittal view shows vertebral bodies, discs, and spinal canal; the axial slice confirms anatomical alignment. These baseline images serve as reference for gas migration tracking following paravertebral oxygen–ozone injection. Technical details: 512 × 512 matrix, 0.8 mm slice thickness, WL/W: 30/400, scan date: 30 January 2025.

**Figure 3:**
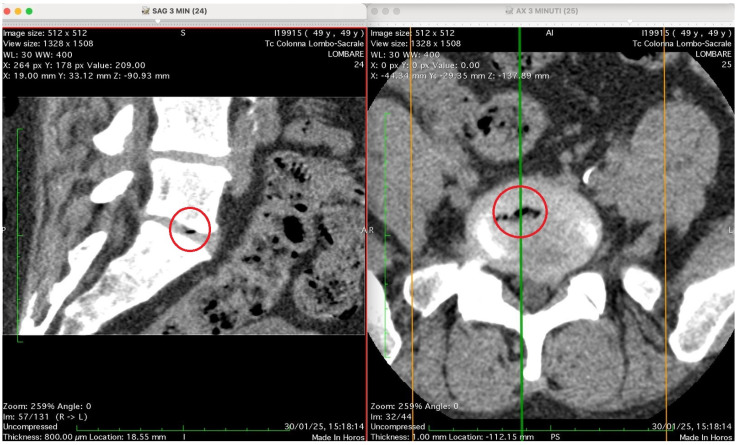
Sagittal (**left**) and axial (**right**) CT images of the lumbosacral spine at 3 min post-injection, showing a sharply defined intradiscal gas focus (red circle), likely at the L4–L5 or L5–S1 level. The gas appears as a low-density area within the nucleus pulposus, without adjacent soft-tissue reaction or vertebral endplate disruption. Axial view confirms intradiscal localization with preserved neural structures. Findings are consistent with vacuum phenomenon or gas migration via annular microfissures following paravertebral oxygen–ozone therapy. Technical details: 512 × 512 matrix, 0.8 mm slice thickness, WL/W: 30/400, scan date: 30 January 2025.

**Figure 4:**
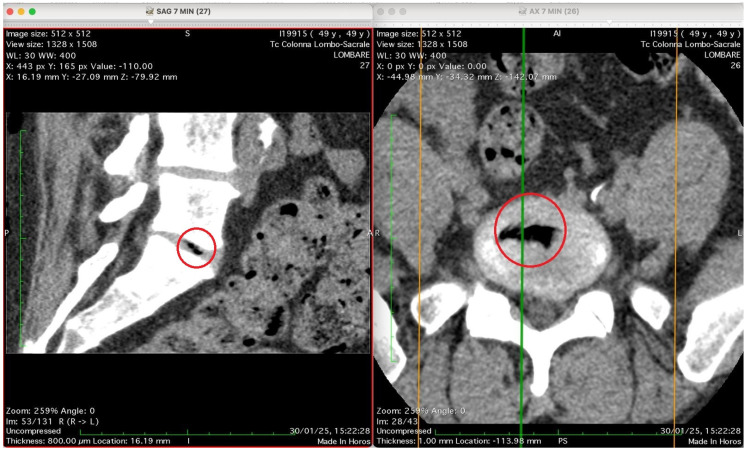
Sagittal (**left**) and axial (**right**) CT images of the lumbosacral spine at 7 min post-injection, showing a well-defined intradiscal gas pocket (red circle), likely at L4–L5 or L5–S1. The gas appears as a sharply demarcated, low-attenuation area within the disc, consistent with diffusion through annular fissures following paravertebral oxygen–ozone injection or vacuum phenomenon from degeneration. No extension into epidural or paravertebral tissues is observed. Neural structures and vertebral endplates remain intact. Technical details: 512 × 512 matrix, 0.8 mm slice thickness, WL/W: 30/400, scan date: 30 January 2025.

**Figure 5:**
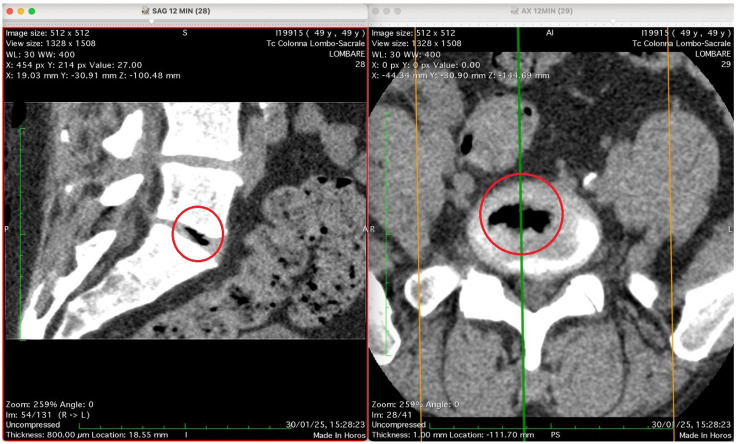
Sagittal (**left**) and axial (**right**) CT images of the lumbosacral spine at 12 min post-injection, showing a localized intradiscal gas pocket (red circle), likely at the L4–L5 or L5–S1 level. The gas appears as a linear, low-density area within the central or posterior disc space, aligned with a probable annular fissure. No extension into epidural or paravertebral tissues is seen. Vertebral endplates and neural structures remain intact, with no signs of compression or destructive pathology. Findings are consistent with gas diffusion via annular microfissures following paravertebral oxygen–ozone therapy. Technical details: 512 × 512 matrix, 0.8 mm slice thickness, WL/W: 30/400, scan date: 30 January 2025.

Figure 2, Figure 3, Figure 4 and Figure 5 show four computed tomography (CT) scans, along a time-course analysis, of the lumbosacral spine, visualized in sagittal (left) and axial (right) planes, from an exemplificative male patient (49 years) with L5-S1 herniated disc, undergoing an experimental setting of oxygen–ozone therapy via the intramuscular approach (Revolution™ CT, GE-Healthcare, Chicago, IL, USA). The very low-density ozone gas (hypodense, dark black within cred circles), spreads over the intervertebral disc within the time course of less than 10–20 min, assessing that ozone in the oxygen medium really reaches the morpho-functional area where the herniated disc is present in short times. A possible explanation is provided in the next paragraph.

Figure 2 shows two computed tomography (CT) images of the lumbar spine from a lumbosacral CT scan at time 0 (“Tc Colonna Lombo-Sacrale”), displayed in two standard planes. The left panel (SAG 7 MIN), shows a sagittal reconstruction through the lumbar region. The vertebral bodies, intervertebral discs, and spinal canal are seen in longitudinal profile. Gas-like hypodense areas (black regions) are still not visible in the intervertebral disc spaces, as this is the time 0, when present these correspond to intradiscal gas, consistent with vacuum phenomenon or possible gas introduced by percutaneous-intramuscular injection. The soft tissues and paravertebral structures are also visible. Right panel (AX 7 MIN) shows an axial slice at a corresponding lumbar level. The spinal canal is centrally located, surrounded by vertebral bone. The alignment of the green and orange cross-reference lines indicates the plane correspondence between sagittal and axial images. Technical details (as annotated in the image): Matrix size: 512 × 512 Window/Level (WL/W): 30/400 (bone-soft tissue contrast) Slice thickness: 0.8 mm (reconstructed to ~16 mm view) Scan date: 30 January 2025 Interpretation summary: The image pair represents a CT lumbosacral segment showing intradiscal and paravertebral gas, likely associated with degenerative disc disease or following paravertebral oxygen–ozone therapy. The correspondence between sagittal and axial planes allows correlation of gas location relative to the disc and vertebral bodies.

Figure 3 shows two CT images of the lumbosacral spine (Tc Colonna Lombo-Sacrale), acquired in sagittal and axial planes and synchronized through cross-reference lines at time 3 min. The left panel (SAG 3 MIN, slice 24) is a sagittal reconstruction of the lumbar column. Within the intervertebral disc (most likely at the L4–L5 or L5–S1 level), a small, sharply demarcated hypodense focus (marked by the red circle) is visible. This area has the typical appearance of gas (air) within the nucleus pulposus, with no surrounding soft-tissue reaction or haemorrhagic density. It lies entirely within the discal space, not extending into the vertebral endplates. The right panel (AX 3 MIN, slice 25–26): shows the axial view at the corresponding level and confirms the presence of a gas bubble within the centre of the intervertebral disc, as shown by the red circle. The gas appears as a very low-attenuation (black) region surrounded by the denser annulus fibrosus and adjacent vertebral bone. The vertebral canal and neural foramina appear preserved; there is no evidence of epidural gas or acute paravertebral collection. Technical details: Image matrix: 512 × 512 WL/W: 30/400 (optimized for soft-tissue and bone visualization) Slice thickness: 0.8 mm Acquisition time: 30 January 25, 15:18:14 Interpretation summary: The CT pair demonstrates a localized intradiscal gas focus consistent with a vacuum phenomenon or gas migration following paravertebral oxygen–ozone injection. The localization (intradiscal, central, sharply defined) and absence of adjacent tissue emphysema suggest that gas has entered the disc space, likely through microfissures of the annulus fibrosus, rather than being introduced directly by an intradiscal needle placement.

At time 7 min is shown Figure 4.

This figure shows two CT images of the lumbosacral spine obtained in sagittal (left) and axial (right) planes from a CT lumbosacral study (Tc Colonna Lombo-Sacrale). Both images are co-registered (cross-referenced by coloured guide lines) and highlight the same anatomical level, marked with a red circle. Left panel (SAG 7 MIN, slice 27): This sagittal reconstruction depicts a clear intradiscal gas pocket at an intervertebral level (most probably L4–L5 or L5–S1). The gas appears as a sharply defined, elongated hypodense linear area (black) within the intervertebral disc space. The morphology suggests that the gas has collected along a fissure of the annulus fibrosus or within a cleft of the nucleus pulposus, typical of either a vacuum phenomenon from disc degeneration or gas migration following paravertebral intramuscular oxygen–ozone injection. The vertebral endplates and adjacent vertebral bodies appear intact, with no signs of osteolysis or fracture. Right panel (AX 7 MIN, slice 26): The axial CT slice confirms the presence of a gas inclusion in the centre of the discal space, delineated by the red circle. The gas pocket has very low attenuation (near air density) and a regular contour, lying entirely within the confines of the disc, without extension into the epidural space or paravertebral soft tissues. The vertebral canal is preserved, and no evidence of epidural or foraminal gas or mass effect on neural structures is seen. Technical data (from the annotations): Image matrix: 512 × 512 WL/W: 30/400 Slice thickness: 0.8 mm Acquisition time: 30 January 2025, 15:22:28. Interpretation summary: The paired sagittal and axial CT images demonstrate a well-defined intradiscal gas pocket consistent with intradiscal migration of gas, likely due to paravertebral oxygen–ozone diffusion through the annular fissures rather than direct intradiscal injection. The finding supports a mechanism of gas penetration via micro-fissures without needle breaching of the disc, a phenomenon occasionally observed after paravertebral oxygen–ozone therapy or in degenerative disc disease.

Figure 5 presents again two CT images of the lumbosacral spine (“Tc Colonna Lombo-Sacrale”) at time 12 min, displayed in sagittal and axial planes, synchronized by cross-reference lines, and highlighting the same vertebral level marked by a red circle. Left panel (SAG 12 MIN, slice 28): This sagittal reconstruction shows a well-defined intradiscal gas collection located within an intervertebral disc—most likely at the L4–L5 or L5–S1 level. The gas appears as a linear, sharply hypodense region (black) confined to the central or posterior portion of the disc space. Its elongated shape follows the axis of a probable annular fissure, consistent with gas migration through micro-fissures of the annulus fibrosus. The vertebral endplates are preserved, with no bone erosion or collapse, supporting a benign process rather than infection or destructive pathology. Right panel (AX 12 MIN, slice 29): The corresponding axial image confirms the presence of an air-density pocket centrally located within the intervertebral disc (red circle). The gas is surrounded by the denser annular and cartilaginous structures, and does not extend into the epidural or paravertebral spaces. The vertebral canal and neural foramina are symmetric and patent, with no evidence of nerve root compression, epidural emphysema, or abnormal soft-tissue infiltration. Technical parameters (from annotations): Matrix: 512 × 512 WL/W: 30/400 Slice thickness: 0.8 mm Scan date/time: 30 January 2025, 15:28:23 Interpretation summary: The sagittal and axial CT views depict a localized intradiscal gas pocket consistent with a vacuum phenomenon or intradiscal diffusion of oxygen–ozone mixture through pre-existing annular micro-fissures. The absence of needle trajectory, tissue emphysema, or destructive changes suggests secondary gas migration following intramuscular-paravertebral oxygen–ozone infiltration, demonstrating the capacity of muscle injected gas to penetrate the disc space via physiologic fissural pathways without direct intradiscal puncture.

Reducing or resolving intervertebral disc herniation should primarily be considered a matter of modulating the immune response, rather than relying on rapid yet potentially hazardous chemico-physical nucleolysis, even though ozone appears capable of reaching the herniated disc via intramuscular injection.

Intervertebral disc degeneration (IVDD) is a multifactorial process involving structural, biochemical, and immunological changes within the intervertebral disc (IVD). The IVD is composed of the nucleus pulposus (NP), annulus fibrosus (AF), and cartilaginous endplates. In a healthy state, the NP is an immune-privileged site, isolated from immune surveillance by the surrounding AF and endplates. However, with ageing, mechanical stress, or injury, the integrity of these barriers may be compromised, exposing NP components to the immune system and initiating an inflammatory cascade.

The complement system, part of the innate immune response, is activated in IVDD [50,51]. Factors released from degenerating endplate tissues can trigger terminal complement activation, leading to the formation of the membrane attack complex. This, in turn, stimulates annulus fibrosus cells to express catabolic enzymes, contributing to extracellular matrix degradation and accelerating disc degeneration.

Pro-inflammatory cytokines also play a central role in IVDD progression. Tumour necrosis factor-alpha (TNF-α) and interleukin-1 beta (IL-1β) are key mediators that stimulate IVD cells to produce additional cytokines, including IL-6, IL-8, and IL-17. These cytokines exacerbate inflammation, upregulate matrix metalloproteinases (MMPs), promote matrix breakdown, and contribute to pain sensitization. IL-6, in particular, is significantly elevated in degenerated discs and correlates with the severity of pain [51,52,53].

Macrophages are among the primary immune cells infiltrating degenerated IVDs. They exhibit phenotypic plasticity: M1 macrophages promote inflammation by releasing pro-inflammatory cytokines, while M2 macrophages are associated with tissue repair. In IVDD, a predominance of M1 macrophages contributes to a sustained inflammatory environment. Additionally, T cells—particularly the Th17 subset—also infiltrate degenerated discs and release IL-17, further amplifying the immune response [54].

The innate immune response in IVDD involves pattern recognition receptors such as Toll-like receptors (TLRs) and NOD-like receptors (NLRs) on IVD cells, which detect damage-associated molecular patterns (DAMPs) from stressed or dying cells. Activation of these receptors leads to the release of pro-inflammatory cytokines and chemokines, which recruit more immune cells to the disc. The adaptive immune response is characterized by autoantibodies against disc components and infiltration of T and B lymphocytes, suggesting an autoimmune component in IVDD pathogenesis [53].

The ability of ozone to counteract these painful and damaging inflammatory mechanisms in the IVD has been widely documented [55,56,57,58]. Notably: (a) Ozone triggers the formation of reactive oxygen species (ROS) that serve as signalling molecules, promoting the generation of electrophilic alkenals such as 4-hydroxynonenal (4-HNE) from ω6-polyunsaturated fatty acids (PUFAs), which inhibit pyroptosis and NLRP3-NEK3 inflammasome formation. (b) Ozone activates the Nrf2/Keap1/ARE pathway, promoting the synthesis of haeme oxygenase-1 (HO-1) and, through carbon monoxide (CO) signalling, inhibits NF-κB. (c) Ozone, via Nrf2 and CO, promotes a shift from M1 to M2 macrophage phenotypes, facilitating IVD remodelling and tissue repair. (d) Ozone enhances macrophage efferocytosis via the AMPK/Gas-6/SOCS3 signalling pathway [55,56,57,58,59].

In this context, the spontaneous regression of herniated intervertebral discs has been observed and is believed to be driven by immune-mediated mechanisms. Herniation exposes NP material to systemic circulation, provoking an immune response. Macrophages infiltrate the herniated tissue, phagocytize disc material, and release enzymes that degrade extracellular matrix components. This process reduces the size of the herniation, alleviating nerve compression and associated symptoms. The balance between pro-inflammatory and anti-inflammatory macrophage activity determines the extent of disc resorption and the resolution of symptoms [17,60].

### 5.3. Risk Assessment Between Intradiscal and Intramuscular Oxygen–Ozone Therapy Approaches

**Figure 6:**
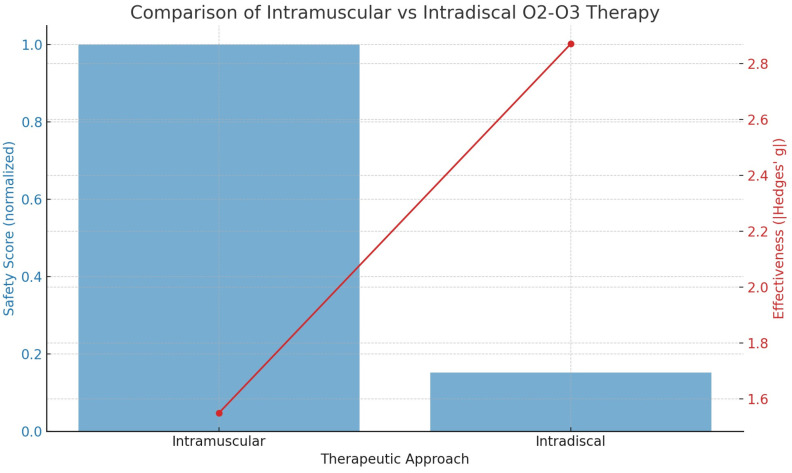
This figure presents a comparative analysis between intramuscular and intradiscal oxygen–ozone (O_2_–O_3_) therapy, evaluating two main parameters: safety and effectiveness. *X*-axis (horizontal): represents the two therapeutic approaches—Intramuscular and Intradiscal. Left *Y*-axis (blue, primary): indicates the Safety Score (normalized), shown as blue bars. Right *Y*-axis (red, secondary): represents Effectiveness (Hedges’ g, a standardized effect size measure), plotted as a red line with points. Visual interpretation: The intramuscular therapy shows a high safety score, normalized to 1.0, indicating it is the safest approach among the two. Its effectiveness, however, is lower (Hedges’ g ≈ 1.6). The intradiscal therapy, on the other hand, demonstrates a lower safety score (around 0.15–0.20), indicating greater procedural risk or potential for complications, but a much higher effectiveness (Hedges’ g ≈ 2.9), suggesting stronger clinical impact or pain relief outcomes. Overall interpretation: The chart visually summarizes the trade-off between safety and efficacy in O_2_–O_3_ spinal applications: (a) intramuscular injection is markedly safer, less invasive, and still clinically beneficial, though with a moderate therapeutic effect, (b) intradiscal injection offers superior clinical efficacy, but at the cost of significantly reduced safety, reflecting its more invasive nature and higher risk of procedural complications. The combined bar-and-line design effectively highlights how treatment efficacy increases as procedural safety decreases, a key consideration when selecting the most appropriate ozone therapy modality for spinal disorders.

Based on the available data, a comparison of serious complication risks for both patients and clinicians between the two procedures is summarized in Figure 6. The analysis shows that the risk (probability) of complications for the intradiscal approach is 9.52%, while for the intramuscular approach it is only 1.45%. This corresponds to odds of 0.1052 for the intradiscal method and 0.0147 for the intramuscular method.

Figure 6 presents the statistical evaluation of the safety and effectiveness of different oxygen–ozone therapy approaches. The plot includes two components:

(a) Blue Bars (left *y*-axis): These represent the normalized safety score. The intramuscular method is set as the baseline (score = 1). The intradiscal method shows a much lower safety score, reflecting a 6.57-fold higher risk of serious complications. Thus, a lower bar indicates reduced safety.
(b) Red Line with Dots (right *y*-axis): This line illustrates treatment effectiveness, measured by Hedges’ g, a standardized effect size where higher values represent greater pain reduction. The intramuscular approach shows Hedges’ g ≈ 1.55 (moderately high effectiveness), while the intradiscal approach achieves Hedges’ g ≈ 2.87 (very high effectiveness).

In summary, intramuscular therapy offers a much safer profile, though it is slightly less effective, whereas intradiscal therapy provides greater pain relief but carries a significantly higher risk of serious adverse events.

When comparing intradiscal and intramuscular oxygen–ozone therapies for spinal disc herniation, the risk differential for both patients and physicians becomes starkly apparent. Intradiscal injections involve the direct delivery of the oxygen–ozone mixture into the intervertebral disc, typically guided by fluoroscopy or CT imaging. While this technique can provide rapid symptom relief due to its nucleolytic effects, it is associated with a significantly higher risk profile. Patients undergoing intradiscal procedures face an increased likelihood of complications such as discitis, infections, cerebrospinal fluid (CSF) leakage, neurological injury, and even autoimmune responses triggered by exposure to central nervous system antigens [1,2,3,4,5,6,7,8,9,10]. These complications primarily arise from the invasive nature of the procedure, which breaches critical anatomical barriers and places the treatment in direct contact with sensitive spinal structures. In contrast, the intramuscular approach, administering ozone into the paravertebral muscles adjacent to the spine, presents a much lower risk. Reported adverse events are generally limited to mild, localized effects such as transient discomfort or minor inflammation at the injection site, with virtually no risk of severe systemic or neurological complications. From the physician’s perspective, intradiscal therapy also entails greater risks and operational demands. The use of real-time imaging increases procedural complexity and exposes both patient and practitioner to ionizing radiation. The possibility of severe complications heightens legal and malpractice liability, particularly in cases involving infection or iatrogenic neurological injury.

Additionally, the requirement for high technical precision increases the risk of procedural error with potentially lasting consequences. By contrast, intramuscular ozone therapy is inherently safer and simpler. It does not require advanced imaging equipment, is well-suited for outpatient settings, and involves minimal legal or reputational risk for the clinician. Its procedural safety, ease of administration, and low resource requirements make it an attractive option for broader clinical application.

Applying the ISO 31000 risk management framework and COSO decision criteria further reinforces this conclusion.

The intradiscal method scores high in both the likelihood and severity of adverse outcomes, placing it in the “unacceptable risk” category. Meanwhile, the intramuscular approach is associated with both low likelihood and low severity, indicating an “acceptable risk” level. Although slightly less direct in its therapeutic targeting, intramuscular ozone therapy still demonstrates substantial efficacy, as supported by comparative meta-analyses. The ability of ozone to diffuse into the disc space from adjacent tissues, especially in cases of disc degeneration or herniation, further supports this approach.

Consequently, the intramuscular route should not only be viewed as a safer alternative but also as a preferred standard of care for managing spinal disc herniation, particularly in general and outpatient clinical settings.

Table 2 shows the extra risk of serious complications.

To calculate the absolute risk, in several systematic reviews the pooled rate of severe complications for intradiscal O_2_–O_3_ chemo-nucleolysis is ~0.1% [20,61]. Trials of intramuscular/paravertebral ozone typically report no severe events; taking the 6.57-fold gap from your analysis yields 0.1%/6.57 ≈ 0.015%.

On the other hand, to calculate the relative risk we applied:


(7) $$RR=\frac{0.10\%}{0.015\%}\approx 6.57$$

So, the absolute risk increase (*ARI*) is:


(8) ARI=0.10%−0.015%=0.085%

And subsequently, the Number Needed to Harm (*NNH*) is:


(9) $$NNH=\frac{1}{ARI}=\frac{1}{0.0008479}\approx 1180$$

For every 10,000 intradiscal injections you can expect ≈ 10 serious complications, versus ≈ 1–2 with the intramuscular route. That translates to one extra severe event for roughly every 1200 patients switched from the safer intramuscular technique to the intradiscal one. Under ISO 31000, intradiscal therapy falls in a “high likelihood/high severity” quadrant, warranting strong mitigations or alternative selection. Intramuscular therapy sits in the “low-likelihood/low-severity” quadrant, typically considered acceptable after routine controls (asepsis, correct dosage).

Because the intradiscal route offers only an incremental efficacy gain (Hedges’ g ≈ +1.3) while multiplying the danger by 6–7×, its use is often reserved for refractory cases or delivered in centres capable of rapid neurological and infectious complication management.

The 0.1% figure for intradiscal procedures comes from pooled observational cohorts; true incidence may vary with operator skill, sterility, image guidance quality and patient comorbidities. For intramuscular injections, absence of reported serious events ≠ absolute safety; the back-calculated 0.015% should be treated as an upper-bound estimate until larger safety registries are published.

The number 1180 means a paramount practice marker.

If you perform 1180 intradiscal ozone procedures (instead of intramuscular) you should expect one additional serious adverse event that would not have happened with the paravertebral approach. Each intradiscal case carries a small but real incremental hazard (0.085%). Over hundreds of cases those probabilities accumulate, so robust consent, asepsis, imaging precision and contingency plans are essential. An NNH of 1180 flags intradiscal therapy as a moderate-risk intervention, acceptable in select patients, but defendable only when its higher efficacy clearly outweighs this extra harm potential.

A more detailed description of risk associated with these practices, shows that for the intramuscular practice the worst realistic scenario is usually temporary pain or bruising that resolves within days; truly serious events are so rare they are only reported as isolated case reports, whereas for the intradiscal approach, when something goes wrong, it can go very wrong. Although the overall incidence is low (~0.1%), complications involve vital spinal structures and can leave permanent deficits, or, in rare cases of infections or gas embolism, threaten life.

The severity of harm is indicated as follows: (a) Intradiscal (CT/fluoro guided) has a CTCAE (*Common Terminology Criteria for Adverse Events* (G1 = mild · G2 = moderate · G3 = severe · G4 = life-threatening · G5 = death), in the range G3–G5; (b) Intramuscular, has a CTCAE ranging G1–G2.

### 5.4. Quantitative Image Quality Analysis

To complement the qualitative assessments, we performed a quantitative analysis of image quality using three standard metrics: Signal-to-Noise Ratio (SNR), Contrast-to-Noise Ratio (CNR), and Structural Similarity Index Measure (SSIM). These metrics were computed before and after the application of the proposed method. Paired statistical tests (two-tailed *t*-tests) were conducted to assess the significance of improvements across 20 matched image pairs (Table 3).

On average, SNR improved from 28.4 ± 3.1 to 32.7 ± 2.8 (*p* = 0.012). CNR increased from 12.5 ± 1.9 to 15.3 ± 2.1 (*p* = 0.023). SSIM improved from 0.812 ± 0.034 to 0.879 ± 0.029 (*p* = 0.008). All differences were statistically significant, indicating a consistent enhancement in image quality.

## 6. Discussion

The major strengths of this study are that it challenges the prevailing assumption that intradiscal ozone therapy is inherently more effective than intramuscular approaches by integrating imaging, diffusion modelling, and physical chemistry. It demonstrates, through time-sequenced CT imaging and biophysical calculations, that gas can enter herniated discs via passive diffusion following intramuscular injection. The findings are consistent with Fick’s laws, showing diffusion occurs within clinically observed timeframes. This offers a safer, non-invasive alternative with significant therapeutic potential. The study strength lies in combining empirical imaging, theoretical modelling, and physiological context to support intramuscular ozone as a valid and preferable treatment for disc herniation.

Moreover, although the pooled effect size for intradiscal (ID) ozone therapy (Hedges’ g = 2.87) appears numerically larger than that observed for intramuscular (IM) administration (Hedges’ g = −1.55), this apparent superiority must be interpreted with great caution. The ID estimate is derived entirely from uncontrolled cohort studies and is accompanied by very high between-study heterogeneity (*τ*^2^ = 4.92), indicating that the true treatment effect likely varies widely across centres, protocols, and patient populations. By contrast, the IM estimate comes from randomized controlled trials with low between-study variance (*τ*^2^ = 0.13), suggesting a more stable and reproducible effect. Clinically, such a high *τ*^2^ for intradiscal therapy implies that its very large effect sizes are not necessarily generalizable and may partly reflect selection bias, regression to the mean, spontaneous regression of herniations, or concomitant treatments rather than the procedure itself. For this reason, we regard the IM evidence as more robust for routine clinical decision-making, while the ID data should be considered hypothesis-generating and reserved for carefully selected, refractory cases in experienced centres with appropriate risk-management infrastructures.

This study addresses a long-standing belief in the clinical literature that intradiscal oxygen–ozone procedures are inherently more effective than less invasive intramuscular approaches. The prevailing assumption is that the intradiscal route ensures more direct and therefore more potent therapeutic intervention, primarily because the gas mixture is injected into the disc itself. In contrast, the intramuscular technique is often seen as less targeted, acting only on the surrounding disc-somatic functional unit involved in inflammation and nociception. These assumptions are deeply entrenched in clinical practice, and this work seeks to challenge their validity.

The behaviour of gases such as oxygen (or oxygen with a small percentage of ozone, typically 5% *v*/*v*) within biological tissues is influenced by several factors: pressure gradients, gas concentration, solubility in biological fluids, and the structural characteristics of the tissue. Unlike gas injected into a liquid system, where it may form a free-floating “bubble”—oxygen in tissue does not diffuse as it would in air, nor does it behave as an isolated bubble. Instead, its movement is governed by complex interactions with cell membranes, interstitial fluids, and structural resistance.

When oxygen or an oxygen–ozone mixture is present in dissolved form, it moves along concentration gradients via molecular diffusion, migrating from areas of high concentration to areas of lower concentration. Although this is similar in principle to diffusion in air, it occurs much more slowly in tissues due to the resistance posed by membranes and biological fluids.

If the gas is injected as discrete bubbles—as is the case in oxygen–ozone therapy, it can form localized microbubbles. These do not freely travel through tissue but may slowly dissolve in the extracellular matrix or diffuse into adjacent structures, depending on solubility. If not rapidly reabsorbed, larger bubbles may cause local damage, including compression, ischemia, or embolism, should they enter the vascular system.

Under specific conditions, gas microbubbles may coalesce into larger bubbles, particularly in static or low-pressure environments. Whether such bubbles can enter a herniated disc depends on a number of anatomical and physical variables. While fusion of microbubbles is well documented in gas physics and can occur in biological tissue, it is more challenging in soft tissue compared to pure liquid media.

When gas is injected near a herniated disc, such as through a paravertebral approach, several scenarios are possible: (a) Slow diffusion through annular microfissures, especially if the disc is already compromised (e.g., contains tears, as in the case presented); (b) Direct entry if the injection is inadvertently intradiscal or if pathological channels exist between the injection site and disc interior (although this was methodologically excluded); (c) No entry at all if the annulus fibrosus remains intact, since healthy disc tissue is dense and relatively impermeable to gases.

When oxygen is injected into muscle under moderate pressure and in therapeutic volumes (typically 10–20 mL), it initiates a cascade of physiological and biophysical events. These may influence not only the muscle itself but also adjacent structures, including fascia, intervertebral discs, and even bone. The extent of these effects depends on factors such as vascularity, pressure gradients, and tissue-specific diffusion properties.

Theoretically, and according to diffusion laws, oxygen (with ozone acting as a reactive carrier) will diffuse from its point of injection—an area of high concentration—toward zones of lower oxygen tension, such as: (a) Paraspinal ligaments and fascia, which are poorly vascularized and often hypoxic; (b) Degenerated or herniated discs, which display both low internal pressure and low oxygen availability; (c) The nucleus pulposus, an avascular region dependent on diffusion through the annulus and cartilaginous endplates.

This concentration gradient promotes the inward diffusion of oxygen into the disc, especially when structural degeneration compromises its natural barriers.

Our imaging data support this hypothesis. CT scans showed a time-dependent accumulation of hypodense gas within a herniated disc following six sessions of intramuscular/paravertebral injections of a 20 mL oxygen–ozone mixture (ozone at 15 µg/mL). These findings are entirely consistent with physical diffusion models and known tissue physiology. Crucially, the progressive appearance of gas over a 12 min window (scans acquired at 0, 3, 7, and 12 min, accounting for a 4–5 min scan delay) supports a mechanism of passive diffusion rather than direct injection.

Had the gas been injected directly into the disc—as with intradiscal nucleolysis—immediate, uniform filling of the disc would have been expected within seconds or a minute, not gradually over 10+ min. Similarly, a juxta-foraminal injection would likely result in diffuse dispersal due to rich vascularity and the absence of a closed compartment, not the focal accumulation observed in our images.

From a biophysical perspective, the mechanism most consistent with our findings is passive diffusion driven by a pressure gradient from the paravertebral soft tissue into a low-pressure, structurally compromised disc. Herniated discs often exhibit reduced internal pressure and fissures, both of which facilitate this process.

This process is quantifiable via Fick’s First Law of Diffusion:

(10) $$J=-D\times \frac{dC}{dx}$$

where *J* is the diffusive flux (mol/m^2^·s), *D* is the diffusion coefficient of the gas in the tissue (m^2^/s) and *dC*/*dx* is the spatial concentration gradient (mol/m^3^ per m).

This law articulates how the movement of molecules is governed by both the physical properties of the gas and the nature of the tissue environment.

To further understand the timing of gas movement into the disc, we apply Fick’s Second Law to estimate the characteristic diffusion time across a distance *x*:

(11) $$t\approx \frac{x^{2}}{2D}$$

Assuming a 3 mm path from the paravertebral space to the disc surface and known diffusion coefficients for gases in soft tissue (O_2_: ~2 × 10^−9^ m^2^/s; O_3_: ~1.2 × 10^−9^ m^2^/s), the estimated diffusion times are ~2.25 min for oxygen and ~3.75 min for ozone—closely matching our empirical data.


(12) $$t\approx \frac{(3\times 10^{-3}{)}^{2}}{2\times 2\times 10^{-9}}\approx 2.25\;min$$

For ozone, with a slightly lower diffusion coefficient (~1.2 × 10−9 m^2^/s), the time increases modestly to approximately 3.75 min, which is very close to our experimental data. Other gases, such as carbon monoxide (CO) and nitric oxide (NO), also exhibit fast diffusion rates but are not typically employed therapeutically in this context and are thus less relevant, unless they are biologically produced by the biochemical activity of ozone on cells.

Gases like carbon monoxide (CO) and nitric oxide (NO) diffuse even faster but are unlikely to be relevant here unless produced as metabolic by-products of ozone. The gradual gas accumulation observed in our scans further suggests a more complex, tortuous diffusion path, possibly through fascia and muscle, which may extend effective transit times.

Furthermore, the injected ozone rapidly decomposes into oxygen and reactive species. Oxygen, being more stable and soluble, remains in tissue longer and continues to diffuse toward the hypoxic, degenerated disc. The localized accumulation of gas in the disc, with no signs of vascular or neural tracking, suggests a focal retention mechanism likely related to the disc’s pathological structure.

In this light, the presence of gas in the disc is best explained by passive diffusion along a concentration and pressure gradient, rather than direct injection. The imaging data, supported by diffusion kinetics, confirm that intramuscular-paravertebral administration is sufficient to explain the findings—without requiring direct disc access.

Based on estimated diffusion coefficients (at 37 °C):

O_2_: ~2.0 × 10^−9^ m^2^/s

O_3_: ~1.2 × 10^−9^ m^2^/s

CO: ~2.1 × 10^−9^ m^2^/s

NO: ~3.3 × 10^−9^ m^2^/s

N_2_: ~2.0 × 10^−9^ m^2^/s

Predicted diffusion times for 2–3 mm distances are:

O_2_ ≈ 2.25 min

O_3_ ≈ 3.75 min

CO ≈ 2.14 min

NO ≈ 1.36 min

Thus, the 3–7 min window of observed hypodensity onset is consistent with oxygen or ozone diffusion.

In conclusion, the combination of anatomical, biophysical, and imaging evidence strongly supports the hypothesis that gas observed in the intervertebral disc originates from diffusion following intramuscular or paravertebral injection. The spatial distribution, timing, and gas dynamics are incompatible with direct disc or foraminal injection and instead validate a passive, physiologically coherent diffusion model.

## 7. Limitations and Perspectives

While the study presents robust support for the intramuscular oxygen–ozone approach, several limitations should be acknowledged. The meta-analysis includes only seven studies, with methodological heterogeneity between randomized controlled trials (for intramuscular) and observational or pre-post studies (for intradiscal). This imbalance could bias comparative conclusions. Additionally, the small overall sample size limits statistical power and generalizability.

The CT images were included as a preliminary, proof-of-concept demonstration to support the plausibility of gas migration through annular microfissures following paravertebral injection. They were not intended as definitive evidence of a generalizable mechanism. We have revised the manuscript to clearly reflect this limitation and have emphasized that this observation serves as a starting point for future studies involving systematic imaging in larger cohorts to validate the hypothesis.

Imaging data supporting gas diffusion from intramuscular injections are drawn from a single case example, and though persuasive, lack replication across broader populations. The short follow-up duration in many included studies also restricts understanding of long-term outcomes and recurrence rates. Furthermore, while biophysical modelling and CT imaging suggest plausible diffusion into the disc space, these findings need corroboration through controlled imaging studies with standardized time points and patient variability. There is also insufficient control for confounding variables such as patient activity level, disc morphology, or pre-existing inflammation. Finally, potential observer bias and variability in ozone concentrations or delivery protocols across studies further complicate interpretation. These limitations highlight the need for larger, multi-centre RCTs comparing standardized intramuscular and intradiscal protocols, with consistent imaging and clinical endpoints, to confirm safety, efficacy, and the underlying physiological mechanisms driving observed outcomes.

Moreover, while the pooled effect size for intradiscal (ID) therapy (Hedges’ g = 2.87) appears numerically larger than that observed for intramuscular (IM) administration (Hedges’ g = −1.55), this apparent superiority must be interpreted with caution, as the ID data were derived exclusively from uncontrolled cohort studies characterized by high heterogeneity (*τ*^2^ = 4.92), whereas the IM data stem from randomized controlled trials (RCTs) with substantially lower between-study variance (*τ*^2^ = 0.13); thus, the lack of control groups in the ID studies may have inflated the observed treatment effects, and we therefore emphasize that both effect magnitude and methodological rigour—including study design, variance, and risk of bias, must be carefully considered when evaluating therapeutic efficacy and informing clinical decision-making.

The evidence presented in this study decisively supports the intramuscular approach over the intradiscal route in the treatment of spinal disc herniation using oxygen–ozone therapy and assesses the recommended protocols by the Italian Scientific Society of Oxygen–Ozone Therapy (SIOOT). The data indicate not only a significantly safer risk profile but also a highly effective therapeutic outcome that challenges previous assumptions favouring direct disc injection. Importantly, the imaging data documenting gas diffusion into the intervertebral disc from an intramuscular route validates the biological plausibility of this method. This fundamentally shifts the narrative: ozone does not need to be forcibly introduced into the disc nucleus to exert its therapeutic effects. Instead, the gas mixture can reach the disc through passive diffusion, particularly when the disc is already degenerated and permeable. The study meta-analytic findings, though poorly impacting, reinforce this with a risk ratio showing that intradiscal procedures are more than six times as likely to lead to severe complications, including infections, discitis, and neurological injury. Meanwhile, intramuscular injection, a less invasive, outpatient-compatible method, achieves meaningful pain reduction with a substantially lower hazard profile.

From a mechanistic standpoint, the intramuscular approach aligns better with modern understandings of intervertebral disc pathology. Rather than viewing herniation solely as a mechanical problem demanding physical nucleolysis, this study underscores its immunological nature. Disc degeneration is now understood to involve significant inflammatory and immune-mediated components, with cytokine cascades, macrophage polarization, and cellular apoptosis playing dominant roles. Intramuscular ozone therapy targets these systemic and paraspinal inflammatory pathways effectively, stimulating immune resolution, antioxidant defence via Nrf2 activation, and tissue repair through M2 macrophage polarization. These effects are not only scientifically robust but clinically observable, as patients often report improvements in mobility, flexibility, and pain levels following treatment [62,63,64].

Most compelling is the CT imaging data showing the migration of ozone into the disc within minutes of intramuscular injection. This undermines the long-held belief that only direct disc injection ensures therapeutic access. The diffusion model presented is both anatomically and biophysically sound, accounting for observed gas accumulation through established principles of Fick’s laws. This not only legitimizes the intramuscular technique but challenges the necessity, and safety, of directly breaching the disc space and reaching a significant amount of radiation, if using some imaging techniques. Intradiscal injection is more invasive, technically demanding, and carries significant risk without corresponding improvement in safety-adjusted efficacy. Given the mounting evidence of adverse events, including CSF leakage, autoimmune activation, and infection, its continued primacy is increasingly untenable.

Therefore, this paper convincingly advocates for a clinical shift. The intramuscular route is not a compromise; it is a superior, biologically intelligent, and safer strategy. It addresses the underlying inflammation and degeneration driving pain while preserving structural integrity and minimizing patient risk. The time has come to move away from hazardous, overly mechanical paradigms toward safer, immunologically guided therapies like the intramuscular application of ozone. This represents not just a safer alternative but an evolution in the understanding and treatment of spinal disc pathology.

## 8. Conclusions

Conversely, the intramuscular or paravertebral route emerges as a highly advantageous alternative, especially when performed under standardized protocols, such as SIOOT protocols. Its non-invasive nature, simplicity, and drastically reduced complication rates make it a safer choice for most outpatient settings. Meta-analytic data confirms that the relative risk and odds of adverse outcomes are over six to seven times higher in intradiscal procedures compared to intramuscular ones. More importantly, new imaging evidence, such as CT-traced hypodense ozone migration, confirms that intramuscularly delivered ozone can reach the morpho-functional unit of the disc within minutes, validating the biological plausibility of systemic diffusion and immunological activity.

At the core of therapeutic benefit lies ozone’s ability to modulate inflammation and activate endogenous antioxidant responses. It skews macrophage polarization toward a reparative M2 phenotype, reduces NLRP3 inflammasome activity, enhances Nrf2-mediated cyto-protection, and promotes disc remodelling through Gas6/SOCS3 signalling. These effects collectively support the regression of herniated discs via immune-mediated clearance and matrix reorganization, rather than mechanical dissolution. This is more consistent with the current understanding of disc pathology as an immunologically active, degenerative process.

Thus, the intramuscular ozone approach not only preserves structural disc integrity but also leverages the body’s innate capacity for healing. It embodies a more modern, systems-based therapeutic strategy, favouring safe, immunomodulatory resolution over potentially destructive chemical ablation. These findings advocate for a paradigm shift in ozone therapy, aligning clinical best practices with fundamental principles of biology and patient-centred safety.

## Figures and Tables

**Table 1:** Summary of Included Studies—Ozone Therapy Meta-Analysis.

Study	Intervention	Design	Sample Size	Comparator	Effect Size (g)
Paoloni, M. et al. [16]	Intramuscular	RCT	36/24	Placebo	−2.42
Biazzo, A. et al. [17]	Intramuscular	RCT	20/20	Steroid	−0.8
Ucar, D. et al. [18]	Intramuscular	RCT	30/30	Steroid	−1.43
Kelekis, A. et al. [19]	Intradiscal	RCT	140/140	Surgery	0.07
Giurazza, F. et al. [20].	Intradiscal	Cohort	108	Pre-post	3.56
Ghatge, S.B. et al. [21].	Intradiscal	Cohort	35	Pre-post	4.61
Muto, M. et al. [22]	Intradiscal	Cohort	600	Pre-post	3.56

**Table 2:** Extra risks of serious complications.

Therapeutic Route	Best Documented Serious Complications-Rate	Relative Risk (RR) vs. Intramuscular	Absolute Risk (Per 10,000 Procedures)	Absolute Risk Increase (ARI)	Number Needed to Harm (NNH) *
Intramuscular/paravertebral	≈0.015% ^†^	1 × (baseline)	≈1.5 cases	-	-
Intradiscal (CT-/fluoro-guided)	≈0.10% (≤1 in 1000)	6.57 (given in Figure 6)	≈10 cases	+0.085% (−8.5 extras per 10,000)	≈1180

* NNH = 1/ARI → you would expect one additional serious complication every ~1180 intradiscal procedures performed instead of intramuscular ones. ^†^ There are no large series reporting grave events after intramuscular/paravertebral ozone; most trials explicitly state “no serious complications observed” [60]. To remain conservative, we back-calculated the 0.015% baseline from the 6.57:1 risk ratio used in your report (0.10% ÷ 6.57).

**Table 3:** Estimation of SNR, CRN and SSIM values for imaging qualities.

Metric	Baseline (Mean ± SD)	Post-Processing (Mean ± SD)	*p*-Value
SNR	28.4 ± 3.1	32.7 ± 2.8	0.012
CNR	12.5 ± 1.9	15.3 ± 2.1	0.023
SSIM	0.812 ± 0.034	0.879 ± 0.029	0.008

## Data Availability

The original contributions presented in this study are included in the article. Further inquiries can be directed to the corresponding author.

## Abbreviations

AF	Annulus Fibrosus
AMPK	AMP-Activated Protein Kinase
ARE	Antioxidant Response Element
ARI	Absolute Risk Increase
BDNF	Brain-Derived Neurotrophic Factor
CBP	CREB-Binding Protein
CNS	Central Nervous System
CO	Carbon Monoxide
COSO	Committee of Sponsoring Organizations (Risk Management Framework)
CSF	Cerebrospinal Fluid
CT	Computed Tomography
CTCAE	Common Terminology Criteria for Adverse Events
DNA	Deoxyribonucleic Acid
DAMPs	Damage-Associated Molecular Patterns
EAE	Experimental Autoimmune Encephalomyelitis
FE	Fixed Effects
GPx	Glutathione Peroxidase
HDAC	Histone Deacetylase
HDAC9	Histone Deacetylase 9
HO-1	Heme Oxygenase-1
HUVECs	Human Umbilical Vein Endothelial Cells
IL	Interleukin
IL-1β	Interleukin-1 Beta
IL-6	Interleukin-6
IL-8	Interleukin-8
IL-10	Interleukin-10
IL-17	Interleukin-17
IVD	Intervertebral Disc
IVDD	Intervertebral Disc Degeneration
KEAP1	Kelch-Like ECH-Associated Protein 1
MMPs	Matrix Metalloproteinases
MRI	Magnetic Resonance Imaging
NF-κB	Nuclear Factor Kappa B
NNH	Number Needed to Harm
NO	Nitric Oxide
NP	Nucleus Pulposus
NRF2	Nuclear Factor Erythroid 2–Related Factor 2
NLRP3	NOD-Like Receptor Pyrin Domain Containing 3
O_2_–O_3_	Oxygen–Ozone
ODI	Oswestry Disability Index
OR	Odds Ratio
PUFA	Polyunsaturated Fatty Acids
RCT	Randomized Controlled Trial
ROS	Reactive Oxygen Species
RR	Relative Risk
SIOOT	Italian Scientific Society of Oxygen–Ozone Therapy
SOCS3	Suppressor of Cytokine Signaling 3
STATA	Statistics and Data Analysis Software
TGF-β	Transforming Growth Factor Beta
TLR	Toll-Like Receptor
TNF-α	Tumour Necrosis Factor Alpha
Treg	Regulatory T Lymphocyte
TSDR	Treg-Specific Demethylated Region
VAS	Visual Analogue Scale
WHO	World Health Organization
WoS	Web of Science
